# Effects of perceived change of urban destination on destination attachment

**DOI:** 10.3389/fpsyg.2022.1022421

**Published:** 2022-11-22

**Authors:** Mei Huang, Xiaojie Yang, Danping Liu, Hedan Fang

**Affiliations:** ^1^School of Management, Xihua University, Chengdu, China; ^2^Research Institute of International of Economics and Management, Xihua University, Chengdu, China; ^3^School of Emergency Management, Xihua University, Chengdu, China

**Keywords:** perceived change, destination attachment, travel experience satisfaction, perceived behavioral control, urbanization of tourist destination

## Abstract

The impact of urbanization on tourism is a widespread macroeconomic concern. However, few studies have explored the impact of destination urbanization on such individual tourist behavior as destination attachment. By developing an urbanization perception scale and analyzing tourists’ destination attachment, this study provides empirical evidence for the micro-impact of urbanization. A sample of 825 repeat visitors of Chengdu, China, was included in the partial least squares-based structural modeling. The results of several tests show that the environmental changes caused by green urbanization positively impact tourists’ destination attachment. This study also examines the explanatory role of tourist experience satisfaction and behavior control in this relationship. The findings suggest that tourists’ experience of urban change is critical for the sticky marketing of tourist destinations.

## Introduction

For historical tourism destinations, change is a dilemma. With any environmental change, novelty and expectation occur alongside uncertainty and dilutions of historical imprint (Singh, 2008). Recently, along with a national development strategy driven by innovation, significant changes have occurred in most urban cities in China. These historical cities employ tourism to ensure continued economic growth; therefore, creating a sticky tourist market is essential to secure a competitive advantage. Moreover, cultivating tourists’ destination attachment plays a vital role in garnering loyalty behavior (Yuksel et al., 2010; Patwardhan et al., 2019), supporting the development of tourism (Kang and Lee, 2018), protecting the destination’s environment (Tonge et al., 2014), and promoting the city (Zhang and Xu, 2019). However, how tourists’ destination attachment is affected when the city changes is rarely discussed by researchers, which is a ture critical issue for destination marketers.

In psychology, destination attachment often describes the emotional bond between self and place (Gross and Brown, 2008; Patwardhan et al., 2019). Prior researchers concur that the emotional bond is an underlying premise that the process of attachment requires a long-term and continuous emotional interaction between the individual and the place, as well as the individual’s positive emotional expression of the place (Williams and Vaske, 2003; Hammit et al., 2006; Lewicka, 2011; Chen et al., 2014; Cao et al., 2021). However, most researchers have examined the causes of destination attachment from a static perspective (Cao et al., 2021) and largely ignored the impacts of the dynamic characteristics.

Some studies ascribe destination image, destination attractiveness, and accumulated satisfactory travel experiences as requisite antecedents to establishing destination attachment (Lee and Shen, 2013; Lee et al., 2015; Tan and Chang, 2015; Xu and Zhang, 2015; Reitsamer et al., 2016; Kim et al., 2018). However, when the environment of the tourist destination changes (i.e., urbanization), tourists’ destination attachment may change due to the re-understanding and reconstruction of the image and attractiveness. Moreover, there are conflicting results on the impact of destination changes on tourism. For example, research by Luo et al. (2015) shows that in provincial Chinese cities, urbanization has a significant positive impact on the development of tourism. In contrast, Cao et al. (2019) believe that when tourists enter an unfamiliar destination, they will reduce their perception of behavior control because of uncertainty, negatively impacting travel intention. Thus, the impact of a change of destination on tourists’ behavior has not yet manifested.

Given the above research gaps, this study aims to explore the impact of the change of urban destination on tourists’ destination attachment, as well as the underlining mechanism. In the past decades of rapid development, compared with the rural areas, China’s cities are also undergoing significant degree of urbanization changes. Meanwhile, as well-known tourist cities can get more repetitive tourists than rural areas, there are also stronger emotional bonds between tourists and the destination. Taking urban destination with stronger market stickiness as the research object, it is more convenient for us to observe the impact of urbanization on destination attachment. This inquiry uses Chengdu, a famous historical and cultural tourist city in China, to explore the impact of urbanization on tourist behavior typical in China and other developing countries. The influence of this perception on destination attachment is analyzed by measuring tourists’ perceptions of urbanization in tourist destinations. In addition, this study verifies the explanatory role of behavioral control perception and travel experience in this relationship. Given the ubiquity of the acceleration of global urbanization, understanding the impact of this change on destination attachment can help marketers develop better strategies and determine whether they are feasible in specific tourism markets.

## Literature review

### The change of tourism cities – Urbanization

Urbanization has been a defining global phenomenon and a key driving force for social and economic development during the past century (Liu et al., 2014; Cai et al., 2020). The economic and policy research literature defines urbanization as the process of transferring people and their ability to work from rural areas to urban areas, accompanied by a national transition from an economy dominated by agriculture to one dominated by secondary and tertiary industries (Deng et al., 2018; Cai et al., 2020). This process requires massive infrastructure investments, such as roads, water, electricity, gas, and communication networks (Chen et al., 2019). Hence, urbanization concentrates industries and populations in and around cities, facilitating the development of economies of scale (Li, 2017). Meanwhile, it brings benefits such as improved employment opportunities, sanitation, income, and access to infrastructure services (Liang and Yang, 2019).

In tourism literature, the urbanization concept is further extended to big cities’ economic structural optimization strategies and infrastructure upgrading to enhance cities’ status in the urban system (Xu and Yeh, 2005; Luo et al., 2015). Leading literature has researched the cultural impact of urbanization on the destination and the tourism industry and the effect of this change on tourist behavior. For example, Loeb and Paredes’ (1991) case study of the Valle de Bravo marketplace in Mexico found that the construction of regional modernization has increased the number of tourists but changed the original environment of the destination and weakened its traditional function. Similarly, Singh (2008) example of Manali in Himachal, Himalaya, is an illustration that the lifestyle convenience brought about by urbanization increased the destination’s attractiveness. However, it inevitably encroached on the original tourism resources and desolated the traditional cultural milieu, thus threatening the sustainable development of tourism.

Luo et al. (2015) analyzed the impact of urbanization on tourism destinations from the development dimension of the tourism economy. This study measured urbanization through macroeconomic indicators such as population, the proportion of service industry, the area of gardens and green, and the length of highways, and analyzed its impact on the income of the regional tourism industry. The results showed that in provincial cities, various indicators positively impact tourism income (Luo et al., 2015).

Prior research concludes: that (1) China’s urbanization represents rapid economic development, reflecting the wider tourism industry (Zhang et al., 2013; Luo et al., 2015), and (2) Dramatic changes over the last decade in population and geographic scope, as well as transportation and other infrastructure, occurred in major Chinese cities (Xiang et al., 2011; Liang and Yang, 2019). However, as the research on urbanization is mainly macroeconomic, it fails to deeply understand micro consumption behavior and its psychological mechanism.

### Destination attachment

Attachment theory initially described the mother–child attachment (Bowlby, 1969; Ainsworth and Bell, 1970; Bowlby, 1971). Scholars extended the notion of attachment to various spatial levels – the actual shape and the specific context of time and space (Hidalgo and Hernández, 2001; Scannell and Gifford, 2010). Subsequent tourism research generally relies on place attachment as an alternative (Cao et al., 2021). However, as place attachment relates to the degree and mode of residents’ social participation and integration into the community (McCool and Martin, 1994), long-term interaction and community attachment do not apply to tourists’ attachment to the destination (Cao et al., 2021).

In tourism literature, Yuksel et al. (2010) consider destination attachment a critical part of the self and investigate the influence of destination attachment on emotions and behavior. Cao et al. (2021) suggest that destination attachment can encapsulate the emotional connection between individuals and specific places (Yuksel et al., 2010; Veasna et al., 2013; Chubchuwong et al., 2015; Sohn and Yoon, 2016; Japutra, 2020; Wang et al., 2020), which will change dynamically with developments before, during and after travel (Cao et al., 2021).

From research on both place attachment and destination attachment, we can see that the degree of visitor attachment is closely related to the physical environment and the interaction between the visitor and the environment (Hammit et al., 2006; Cao et al., 2021), which infers the impact of environmental changes on destination attachment. In the visitor-destination interaction process, destination image, destination attractiveness, visitor involvement, as well as accumulated satisfactory tourism experiences are important antecedents for establishing such emotional connections (Lee and Shen, 2013; Lee et al., 2015; Tan and Chang, 2015; Xu and Zhang, 2015; Reitsamer et al., 2016; Kim et al., 2018). Meanwhile, the nature of traveling motivation, where travelers seek a degree of change, novelty, escape, exploration, sensation, and variety in unfamiliar or less familiar environments (Wymer et al., 2010; Assker and Hallak, 2013; Fuchs, 2013), also suggests that the changes of destination may positively impact destination attachment. In addition, Luo et al. (2015) show that urbanization has a significant positive impact on tourism development in provincial Chinese areas.

However, some studies have shown that changes in the tourism environment may adversely affect destinations, especially historical and cultural cities. For example, Singh (2008) proposed that the process of urbanization would encroach on the original location of tourism resources and weaken the traditional cultural atmosphere, thus threatening the development of the tourism industry. Related studies on the preference of repeat tourism show that repeat tourists strongly desire to enjoy the same experience and activities as previous tourism trips (Crouch et al., 2014). Furthermore, Cao et al. (2019) and Yang et al. (2022) found that when tourists enter an unfamiliar environment, their perceptions of behavioral control diminish due to uncertainty, negatively impacting their willingness to travel.

Overall, recent research suggests that destination attachment should link to the dynamic nature of a destination. Changes in a destination’s environment can influence destination attachment. This is because the dynamic change of destination will reflect tourists’ perception of this change and the interaction experience with the destination, thus affecting the destination attachment (Cao et al., 2021).

## Hypothesis development

Changes in the appearance of Chinese cities caused by urbanization mainly manifested as population growth, the expansion of geographical scope, and large-scale investment in infrastructure (Cai et al., 2020). In addition, since 2012, China has begun to emphasize the concept of green development (Yang, 2013). Urban development now integrates green and sustainable goals to solve the environmental pollution caused by rapid urbanization and begin achieving results (Yao et al., 2021).

Considering the changes brought by green urbanization to Chinese cities in the past 10 years, the experience of tourists mainly reflects the optimization of the environment, the facilitation of infrastructure, and the intelligent convenience assessments of services. Reitsamer et al. (2016) observed that because consumers form cognitive assessments of their experiences based on their response to the environment, these perceptions are stored as psychological representations and retrieved to form attachments to the environment. Moreover, Reitsamer demonstrated that accessibility and amenities can help tourists form a positive attitude toward the destination, thereby strengthening their destination attachment. Therefore, when tourists perceive the changes in the destination in the above aspects, it can be inferred that their destination attachment will be stronger due to the better experience gained during the interaction with the city. Hence, the following hypothesis is proposed:

*H1*: The stronger the tourists’ perceived changes of the destination, the stronger their positive attachment to it.

Under the influence of urban changes, on the one hand, the construction of a transportation network would improve the accessibility of the destination. At the same time, the increase in amenities and high-quality services provided by urbanization indicates the increase in the ability of destinations to meet the needs of tourists (Vengesayi, 2003). These changes are important conditions to support tourists’ unforgettable destination experience and form the attraction of the destination (Cracolici and Nikamp, 2008; Reitsamer et al., 2016). On the other hand, the application of new information and intelligent technologies in urban infrastructure and services also indicates the changes in the quality of the tourist experience (Pai et al., 2020). Pai et al. (2020) show that the smartness of tourism infrastructure will help to provide tourists with satisfaction in the tourism experience so that tourists can obtain happiness during the traveling process. Therefore, the satisfaction of the tourism experience can explain the positive impact of urban change perception on destination attachment:

*H2*: Tourist experience satisfaction plays a mediating role in the relation between the destination change perception and destination attachment. Specifically, destination change perception will positively affect tourists’ satisfaction of experience, thus positively impacting destination attachment.

Another alternative explanation is that when the appearance of the destination changes, resulting in the individual’s cognition of the destination no longer familiar, it will weaken the safe and stable psychology conducive to the formation of destination attachment (Bott et al., 2003). In other words, the change of destination will cause tourists to perceive that the destination is inconsistent with their previous experience. Furthermore, expanding the geographical scope of the destination will increase the difficulty of travel, which will cause individuals to form factors that hinder their travel activities and reduce the level of behavior control (Lee and Tussyadiah, 2012). Han et al. (2011) and Quintal et al. (2010) showed that a low level of perceived behavior control would lead to the formation of low travel intention. Cao et al. (2019) showed that a low level of perceived behavior control would weaken the level of destination attachment. Therefore, perceived changes in the urban destination may harm destination attachment by reducing tourists’ perceived behavior control.

*H3*: Perceived behavior control mediates the relationship between urban change perception and destination attachment. Specifically, urban change negatively affects the level of perceived behavior control, thus negatively impacting destination attachment.

**Figure 1:**
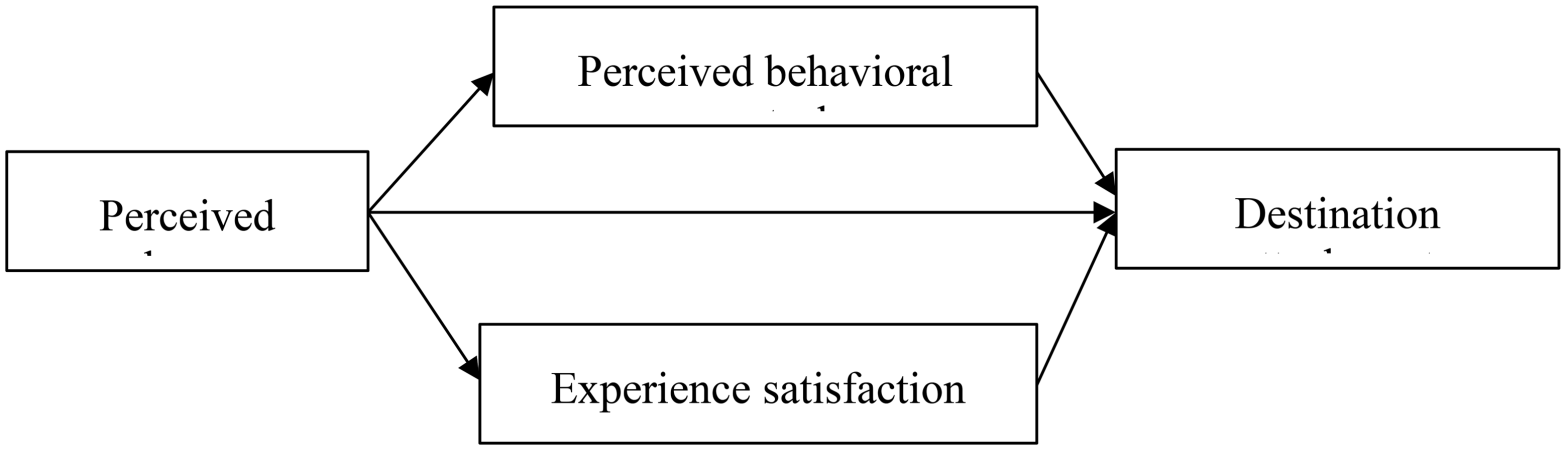
Conceptual framework for perceived change and destination attachment.

Figure 1 demonstrates the conceptual framework covering the three research hypotheses discussed in this section.

## Research methodology

### City selection

Undoubtedly, Chengdu, the capital of Sichuan province, is one of the most changed cities in China. The administrative area of Chengdu gradually expanded after the founding of New China. From 1990 to 2020, Chengdu has changed from seven districts to a city governing 12 districts and eight counties. Populations have grown from 91.95 million to 151.97 million from 1990 to 2020. The land area has grown from 12,121 square kilometers to 14,335 square kilometers.

In the past decade, great changes have taken place in Chengdu city. Tianfu’s new area was established in 2010 and approved as a national new area in 2014, marking the urban pattern of Chengdu from “single core driven” to “dual core co-prosperity.” In 2012, the grand goal of Chengdu’s “hundred-mile city axis” was put forward, and a “giant dragon” connecting the Chengdu Plain Economic Zone for nearly a hundred miles was impressively displayed on the big “picture” of Chengdu. In 2017, the scope of Chengdu’s five major sub-districts, namely, “East expansion, South expansion, West control, North reconstruction, and middle optimization,” was delimited, starting with the millennium change of the urban pattern from “two mountains and one city” to “one mountain and two wings.” In the same year, the Chengdu municipal government approved the request for instructions on naming the ring road by the Municipal Civil Affairs Bureau, and Chengdu became the second city with the Sixth Ring Road in China. In just 20 years, Chengdu has developed from an “inner city” to a super city.

Meanwhile, Chengdu is also a famous historical and cultural city in China and the capital of gastronomy in the world, with rich tourism resources, such as Dujiangyan, Wuhou Temple, Du Fu Thatched Cottage, and other places of interest. As one of the leading tourist cities in China, Chengdu is a highly attractive and popular tourist destination where destination attachment strength is particularly robust. According to the statistical yearbook of Chengdu City in 2021, under the pressure of COVID-19, the total tourism income of Chengdu City reached over $45 billion, the number of domestic tourists reached 203.95 million, and international tourists exceeded 191.9 million. In short, Chengdu fits the research context and is a representative tourism destination for investigating tourists’ perceptions of change and destination attachment, with indicative implications for numerous tourism destinations worldwide.

### Measurement

Items were developed for the change perception of the destination based on the measures of urbanization in the research of Luo et al. (2015), which includes GDP *per capita*, non-agricultural population proportion, hospital beds, and amount of garden and green space. Considering the indicators that tourists can involve at the perception level, the change in perception of the destination includes seven items, changes in the overall environment, changes in geographical scope, changes in population, intelligent tourism services, and facilitation of transportation networks, the integrity of infrastructure, and the informatization of infrastructure. The four dimensions of destination attachment measure (Kyle et al., 2005; Chen et al., 2014) have repeatedly been proven to have high reliability and validity in different studies (Morhart et al., 2009; Chen and Dwyer, 2017) and were applied in this study. The scale of travel experience satisfaction from Yuksel et al. (2010) and the four items of behavioral control perception from Cao et al. (2019) and Pangaribuan et al. (2021) were adopted in this study. The questionnaire consisted of 27 items measured on a 7-point Likert scale.

### Sampling

Xu and Zhang (2015) show that foreign tourists visiting China are weaker than domestic tourists in terms of destination attachment. In addition, there are obvious differences in urban live and consumption between China and the other countries. Many foreign tourists who only travel for a short time may not fully experience the upgrading of infrastructure in Chinese cities, such as transportation systems, payment system and other software. Therefore, this study intends to determine the impact of urbanization changes in tourist destinations on tourists with profound experience through a questionnaire survey of domestic tourists.

An online survey *via* Credamo – an online research platform in China^1^—was carried out between July and August 2022. All the respondents are non-residents and visited the city at least twice from 2012 to 2022. Therefore, we set the IP of the participants out of Sichuan Province, and asked the total visit times since 2012 in the questionnaire in order to screen the respondents. In total, 1,186 questionnaires were randomly collected. Among them, due to 76 respondents are residents, 283 respondents visited the destination less than 2 times, and 2 respondents failed to pass the attention check, these 361 respondents were excluded from the data set. Of which 825 questionnaires were valid for the data analysis (valid rate of 65.56%).

### Procedure

This study applied partial least squares (PLS)-based structural equation modeling (SEM) approach to test the research model and hypotheses. PLS is acknowledged for its ability to process complex models with a limited sample size (Henseler et al., 2009; Hair et al., 2012). Since the full structural model in this study encompassed 49 paths with eight latent variables (including 1 s-order construct) on a sample size of 825, PLS-SEM was deemed appropriate. This study used Smart PLS 3.0 and performed a standard PLS algorithm (1,000 iterations and a stop of criterion of 10–7), and assessed the significance level of the estimates based on 5,000 bootstraps. The sample statistics were considered significant if they were significant above the 95% confidence level (Perezgonzalez, 2015; Jung et al., 2019).

### Descriptive analysis

The demographics of the sample are illustrated in Table 1. Of the 825 survey participants, 65.4% were female, with an average age of 29.1, (standard deviation is 8.42), and more than half had a university-level education. Additionally, 37.41% of the participants lived in east China. On average, each respondent visited Chengdu 4.71 times (standard deviation is 4.32) in the last 10 years.

**Table 1:** Descriptive statistics.

Sample size (*n*)	825
	Mean (SD)
Age	29.1 (8.42)
Visits to Chengdu	4.71 (4.32)
Gender	%
Male	34.3
Female	65.4
Education	%
Graduate degree or higher	13.45
Bachelor’s degree	69
Junior college	11.4
High school or less	5.94
Permanent residence	%
Northeast China	4.72
Northwest China	5.81
Southwest China	5.09
Central China	16.46
East China	37.41
North China	15.62

### Exploratory factor analysis for perceived change

From the data of the seven items of perceived change, it can be seen that except for changes in geographic scope (mean = 4.42, *sd* = 1.46), it can be generally felt the significant changes in Chengdu’s environment (mean > 5). To check the validity of the perceived change scales, exploratory factor analysis (EFA) was conducted in this research. The appropriateness of factor analysis on perceived change items (KMO = 0.823, Bartlett’s test of sphericity = 1437.14, *df* = 21, *p* < 0.001) showed that the use of EFA was suitable (Hair et al., 1998). The pattern matrix from the EFA for the perceived change items indicated a two-factor solution (Table 2). However, the Cronbach’s alpha of the second factor was less than 0.7, which indicates a low degree of internal consistency of the items. Furthermore, factor loadings on both factors of total environmental change are all less than 0.5, and only factor 1 was retained at the end.

**Table 2:** Exploratory factor analysis results of perceived change items.

Perceived change item	Mean	SD	Cronbach’s *α*	Factor 1	Factor 2
PC1			0.773		
CTS – The degree of tourism service intellectualization	5.72	0.99		0.713	
CTr – The convenience of transportation network	5.91	0.96		0.769	
*CF* – The completeness of infrastructure and facilities	5.72	0.93		0.724	
CI – The degree of informatization of infrastructure and facilities	5.79	0.95		0.743	
CT – Overall environmental change	5.41	1.03		0.489	
PC2			0.657		
CG – Geographical range of the city	4.42	1.46			0.858
CP – Population	5.17	1.2			0.808

### Confirmatory factor analysis

CFA was conducted *via* a structural model in AMOS to further validate and refine the factors. Items with low factor loadings (using the cut-off values above 0.6) were first deleted from the model. Then, for the variables with AVE < 0.5 or CR < 0.7, the items with lower coefficient were gradually removed until the AVE reach above 0.5 and the CR reach above 0.7. According to the results of CFA (Table 3), all AVEs were above 0.50, CR was between 0.711 and 0.897, and the correlation coefficient among each latent variable was less than the square root of the corresponding AVE (Table 4; Fornell and Larcker, 1981), indicating convergent validity and discrimination validity of the constructs (Hair et al., 1998).

**Table 3:** Results of CFA.

Measurement items	Mean	SD	Standardized *β* coefficient (for full items)	AVE, CR (for full items)	Standardized *β* coefficient (for remaining items)	AVE, CR (for remaining items)
Perceived Change – PC1				0.416, 0.779		0.552, 0.711
CTS – The degree of tourism service intellectualization*	5.72	0.99	0.579			
CTr – The convenience of transportation network*	5.91	0.96	0.629			
*CF* – The completeness of infrastructure and facilities	5.72	0.93	0.720		0.738	
CI – The degree of informatization of infrastructure and facilities	5.79	0.95	0.704		0.748	
CT – Overall environmental change*	5.41	1.03	0.577			
Perceived change – PC2*				0.490, 0.665		
CG – Geographical range of the city*	4.42	1.46	0.717			
CP – Population*	5.17	1.2	0.695			
Place identity – PI				0.514, 0.760		0.515, 0.761
PI1 – I feel visiting Chengdu is part of my life	4.32	1.41	0.732		0.747	
PI2 – I identify strongly with Chengdu	5.71	1.05	0.698		0.702	
PI3 – Visiting Chengdu has a special meaning in my life	5.31	1.25	0.719		0.702	
Place dependence – PD				0.509, 0.804		0.561, 0.792
PD1 – I like visiting Chengdu more than any other city	5.42	1.16	0.750		0.744	
PD2 – For me, Chengdu cannot be substituted by other urban destinations*	5.33	1.27	0.625			
PD3 – Chengdu can meet my needs more than other cities	4.93	1.11	0.792		0.799	
PD4 – For the activities that I enjoy most, the settings and facilities provided by Chengdu are the best	4.68	1.24	0.675		0.700	
Affective Attachment – AA				0.648, 0.846		0.707, 0.828
AA1 – Chengdu means a lot to me*	5.15	1.29	0.772			
AA2 – I am very attached to Chengdu	4.89	1.40	0.827		0.846	
AA3 – I have a strong sense of belonging for Chengdu	4.65	1.45	0.814		0.835	
Social bonding – SB				0.524,0.763		0.524, 0.763
SB1 – I have some connection with the local residents of Chengdu	4.79	1.60	0.557		0.555	
SB2 – I feel like the employees and local residents, which greatly enhanced my experience	4.57	1.38	0.775		0.780	
SB3 – I have a special connection with those people who like visiting Chengdu	4.68	1.45	0.813		0.810	
Perceived behavioral control – BC				0.445,0.710		0.815, 0.897
CON – The degree of control over tourism activities in Chengdu*	4.67	1.31	0.126			
CHA – The challenge level of tourism activities in Chengdu*	3.99	1.43	0.423			
ANXI – The anxiety of tourism activities in Chengdu	2.56	1.38	0.894		0.794	
RESTL – The restlessness of tourism activities in Chengdu	2.44	1.34	0.886		1.000	
Travel experience Satisfaction – TE				0.486,0.739		0.571, 0.727
COM – The comfortableness of the tourism environment in Chengdu	5.74	1.00	0.682		0.751	
SAT – The satisfaction of tourism experience in Chengdu	6.04	0.87	0.700		0.760	
PLE – The pleasantness about the trip in Chengdu*	5.83	1.05	0.708			

*Item deleted in PLS because of a low factor loading or with multiple intra-item correlations. CR and AVE for each variable were calculated based on the remaining items.

**Table 4:** Discriminant validity and criterion-related validity.

	PC	PI	PD	AA	SB	BC	TE
PC	**0.743**						
PI	0.431	**0.718**					
PD	0.372	0.690	**0.749**				
AA	0.330	0.705	0.706	**0.841**			
SB	0.352	0.618	0.650	0.664	**0.724**		
BC	−0.061	0.004	0.009	0.047	0.052	**0.903**	
TE	0.401	0.487	0.464	0.431	0.420	−0.166	**0.756**

Square root of AVE is shown on the diagonal of the matrix in bold; inter-construct correlation is shown off the diagonal.

### Results of PLS-SEM

**Figure 2:**
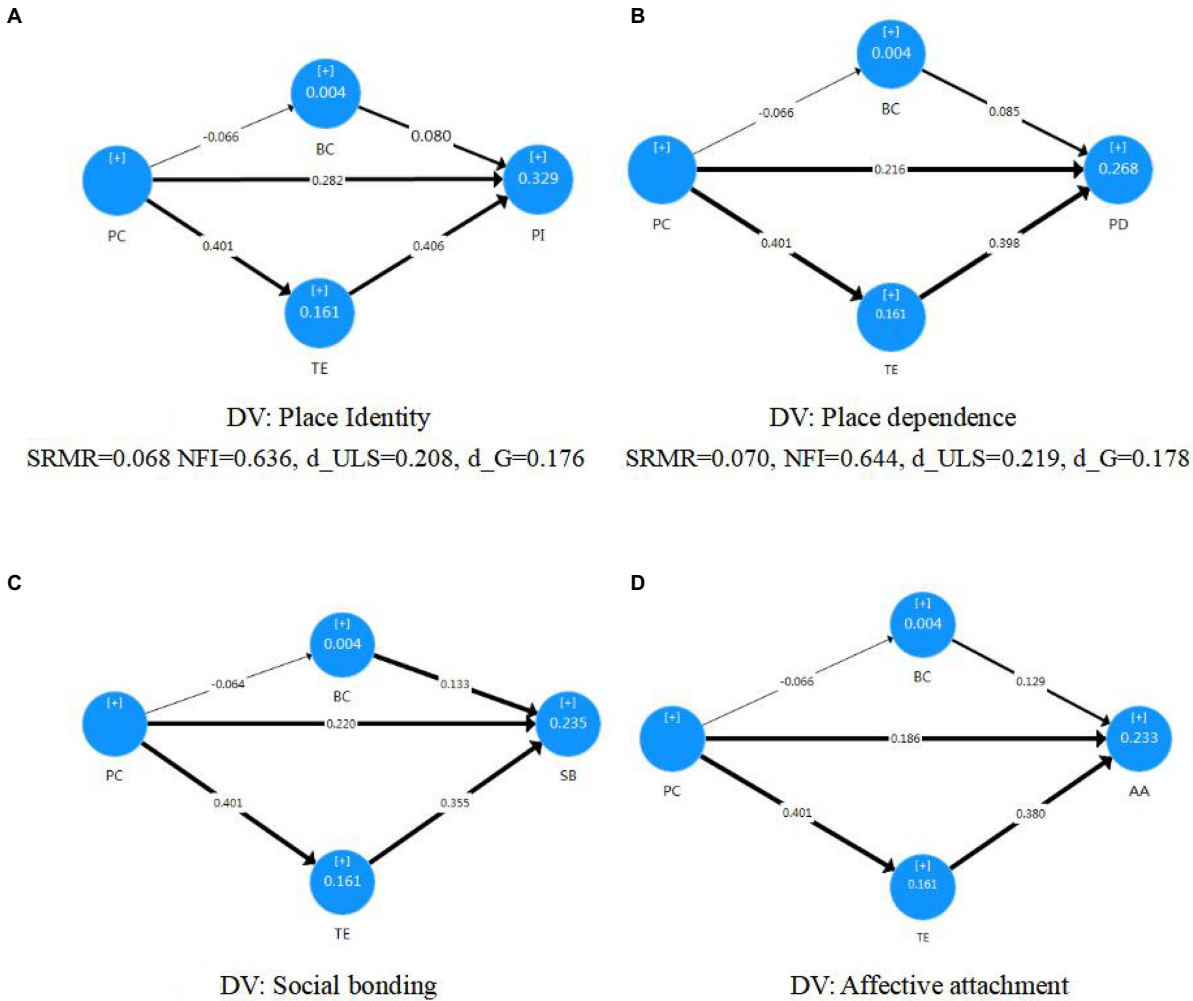
Structural models of relationships with standardized regression coefficients and *R*2 values between perceived change and destination attachment: **(A)** Place identity, **(B)** place dependence, **(C)** social bonding, **(D)** affective attachment (bold paths are significant at *p* < 0.05 or *p* < 0.01 level, numbers in the circles are *R*2 values).

The four destination attachment dimensions and each category of perceived change, travel experience, and behavioral control were placed into separate structural models to verify the effects of perceived change of destination. Table 5 summarizes the path coefficients of the relationships in the tested inner models. Significance levels are calculated on a 5,000-time bootstrapping (Hair et al., 2014). Figure 2 shows the path coefficients and *R*2 values. The SRMR values from the four models are between 0.061 and 0.070, indicating an acceptable model fit.

**Table 5:** Inner model evaluation (hypotheses testing).

Hypotheses	Tested effects	DV: PI	Tested effects	DV: PD	Tested Effects	DV: AA	Tested effects	DV: SB
		*β*-value		*β*-value		*β*-value		*β*-value
	Direct effect							
Hypothesis 1 supported	PC→PI	0.282***	PC→PD	0.216***	PC→AA	0.186***	PC→SB	0.220***
	PC→TE	0.401***	PC→TE	0.401***	PC→TE	0.401***	PC→TE	0.401***
	PC→PR	−0.066^N.S^	PC→PR	−0.066^N.S^	PC→PR	−0.066^N.S^	PC→PR	−0.064^N.S^
	PR→PI	0.08**	PR→PD	0.085**	PR→AA	0.129***	PR→SB	0.133***
	TE→PI	0.406***	TE→PD	0.398***	TE→AA	0.38***	TE→SB	0.355***
	Indirect effect							
Hypothesis 2 supported	PC→TE→PI	0.163***	PC→TE→PD	0.16***	PC→TE→AA	0.152***	PC→TE→SB	0.142***
Hypothesis 3 rejected	PC→PR→PI	−0.05^N.S^	PC→PR→PD	−0.006^N.S^	PC→PR→AA	−0.008^N.S^	PC→PR→SB	−0.008^N.S^

*p < 0.1, **p < 0.01, ***p < 0.001, N.S., not significant. The bold numbers are significant at *p* < 0.05 or *p* < 0.01 level.

For the direct effects of perceived change of the destination, place identity (*β* = 0.282, *p* < 0.001), place dependence (*β* = 0.216, *p* < 0.001), social bonding (*β* = 0.220, *p* < 0.001), and affective attachment (*β* = 0.186, *p* < 0.001) were significantly positively affected. Therefore, Hypothesis 1 is supported.

Fort the perceived travel experience satisfaction, perceived change also had a significant positive impact (*β* = 0.401, *p* < 0.001). However, although perceived change showed a negative effect on perceived behavioral control (*β* = −0.066, *p* > 0.05), it turned out to be insignificant. As a result, the indirect effects of the perceived change *via* travel experience satisfaction on the four dimensions of destination attachment were significant, while the indirect effects *via* perceived behavioral control were insignificant. Hence, Hypothesis 2 is supported, while Hypothesis 3 is rejected.

## Discussion

This research provided crucial insight into the relationship between a perceived change of destination and destination attachment. To answer the research question, this research provides two explanations. One is that urbanization may lead to tourists’ perception of the risk of losing control of behavior, thus reducing tourists’ destination attachment. The second is that urbanization can provide a more convenient and satisfying travel experience, thereby increasing tourists’ destination attachment.

In this study, destination attachment is divided into four dimensions, and the three hypotheses are verified by testing the impact of destination change perception on these four dimensions. First, consistent with Hypothesis 1, the results provide evidence that a perceived change of destination has a positive impact on destination attachment. The items of perceived change reflect changes in environmental factors, such as the completeness and informatization of the infrastructure and facilities, rather than tourism resource factors. Therefore, this result indicates that changes in the destination’s environment, such as the improvement of urban infrastructure in the process of urbanization, have a positive effect on tourists’ destination attachment.

Specifically, the positive impact of perceived change on place identity shows that urbanization can increase tourists’ sense of identity with the destination, enhance the value and significance of the destination to tourists. The positive impact of perceived change on place dependence indicates that the urbanization can increase tourists’ preference for the destination. The positive effect of perceived change on affective attachment shows that the urbanization can increase tourists’ emotional connection and sense of belonging to the destination. Finally, the positive impact of perceived change on social bonding indicates that the urbanization can enhance tourists’ social contact with the destination community.

Second, the results also demonstrate that the improvement of tourists’ travel experience is an effective explanation for the positive impact of Chengdu’s environmental changes. This result complements research by Singh (2008) and Luo et al. (2015) on the impact of urbanization on tourism. At the micro psychological level, among the many environmental changes brought about by urbanization, tourists are obviously more sensitive to the convenience of tourism infrastructure, which is closely related to their tourism activities. Consistent with the research results of Reitsamer et al. (2016), when urbanization invites a more comfortable tourist experience for visitors, they will have a greater stickiness to the tourism destination (i.e., place identity, place dependence, affective attachment, and social bonding). This finding also shows the success of Chengdu’s green urbanization development in the past decades.

Third, the findings showed that an alternative explanation for the mediating role of behavioral control was not established. Although environmental change had a negative effect on behavioral control, this effect was not significant. The application of infrastructure information and intelligent technology is more conducive to making tourists’ travel activities more convenient than making their actions more difficult to control. Meanwhile, since the two items of behavioral control reflect the level of anxiety and uncertainty about tourists’ traveling activities in the destination, their positive impact on destination attachment well reflects the motivation of pursuit of excitement and novelty in tourism behavior. This result shows that changes in the tourism environment are more likely to bring new experiences for tourists, and the challenges brought by this experience may instead promote tourists’ destination attachment.

Although the negative impact of perceived change on behavioral control is not significant, this result suggests that urbanization does not bring travel anxiety to tourists, but can improve their perception of behavior controllability. This may be due to the similarity of infrastructure upgrades and improvements involved in urbanization changes across most cities in China. As a result, tourists are no stranger to the experience in such a tourist city, especially the experience of the use of urban infrastructure. The highly controllable perception of traveling behavior (i.e., familiar traveling experience) makes the novel experience, one of the important motivations of tourism, not satisfied (Wymer et al., 2010), which leads to the reduction of destination attachment (Babu and Bibin, 2004; Jang and Feng, 2007; García et al., 2012). This conclusion infers that the urbanization of the sample city—Chengdu currently conducted has a negative impact on tourism behavior, that is, the urbanization that leads to the similarity between tourist destination and other cities will reduce tourists’ destination attachment.

### Management implications

The results of this study provide evidence for the micro-psychological effects of urbanization on tourism destinations. Accordingly, the impact of this study on tourism city managers is threefold. First, destination managers can consider the positive impact on tourists’ sticky behavior by taking advantage of the convenience of urban infrastructure, particularly soft facilities. That is, destination planners must consider improving the ease of use of infrastructure when carrying out the urbanization improvement of the destination, especially promoting the application of new technologies and artificial intelligence. The main purpose of applying these technologies is to improve functions such as the life and travel convenience of visitors.

Second, to strengthen the city’s sticky tourist market, destination marketers may use the comfort and novelty of tourism infrastructure to reflect changes in the city rather than only show the change in the city’s appearance. For example, the theme of park city development in Chengdu shows the optimization of the urban environment and brings a better ecological environment experience for tourists. Compared with the overall changes in the city, this type of detail is more conducive to promoting the destination attachment of tourists. Although this study does not provide sufficient evidence, excessive changes in the city’s appearance may invite obstacles to tourists’ travel.

Third, the result of Chengdu’s urbanization is a good example of China’s adoption of green development concepts. The results of this study also show that the urbanization of Chengdu positively contributes to the formation of a sticky tourism market. Therefore, when the progress of urbanization is inevitable, destination managers should adhere to the green development theory to avoid adverse effects on the tourism market. Meanwhile, according to our research, urbanization may lead to similarities between tourist destination and other cities, thus reducing the tourism experience. Therefore, when carrying out urbanization, tourist destinations need to pay attention to maintain the difference with other cities and ensure the novel experience of tourists.

Finally, for the problem of over-urbanization, based on the results of this research, our suggestion is that we must restore or redevelop unique tourism resources for the destination. By bringing novel and comfortable tourism experience to tourists, the destination can be rebuild a sustainable tourism market.

## Conclusion

This article addressed the unexplored questions of how an environmental change (i.e., urbanization) in the destination influences tourists’ destination attachment. The conceptual contribution of this study lies in the development of a scale of tourists’ perception of destination urbanization and the establishment of a relationship between urbanization and destination attachment through a structural model. Thus, we expand our understanding of the impact of tourism destination urbanization on the individual behavior of tourists.

To date, prior researches have considered negative impact to tourist behavior by COVID-19, such as travel avoidance (Zheng et al., 2021), and directly cutting off the possibility of destination attachment (Liu and Mair, 2023). However, from the view of urbanization, the global pandemic of COVID-19 has promoted the upgrading of emergency systems in most cities to respond to public health events. In China, this emergency system has become standardized, which makes the emergency systems of all cities in the country highly consistent. Based on the results of this study, although the upgrading of this highly consistent urban emergency system has increased the traveling cost of tourists, it may also reduce the barriers to travel due to operational familiarity, so as to maintain tourists’ destination attachment.

However, this study only makes a preliminary exploration of the psychological perception scale of urbanization according to the previous literature. First, the content involved is limited. In the future, more in-depth research can be conducted on the psychological scale of environmental change, such as increasing the perception survey of economic, ecological, community, and other related environmental factors, to obtain a more comprehensive impact of environmental change on tourism psychology. Second, given the impact of the change, this study used the methodology of tourists’ self-reporting to collect and analyze the data. In the future, more objective empirical research can be carried out through a comparison before and after the change. In addition to the excessive urbanization pointed out in the previous literature, which leads to the unsustainable development of tourism, this study finds that urbanization will lead to the similarity of tourism cities. This conclusion indicates that there may be different types of urbanization, and tourists have psychological and behavioral responses to these changes. At present, the related research is very limited. Therefore, it is necessary to conduct in-depth research on the theoretical framework construction of destinations urbanization as well as empirical researches on different tourist cities in the future.

## Data availability statement

The original contributions presented in the study are included in the article/supplementary material, further inquiries can be directed to the corresponding author.

## Author contributions

MH reviewed the literature, proposed the research model, designed the study, and conducted the experiment. XY collect data and analyze data. DL conducted the literature search, drafted the manuscript, and edited it. HF participated in the edited. All authors discussed, finalized, and approved the manuscript for publication.

## Funding

This work was supported by the Innovation Funding of Research Institute of International Economics and Management, Xihua University, Funding number 20210020.

## Conflict of interest

The authors declare that the research was conducted in the absence of any commercial or financial relationships that could be construed as a potential conflict of interest.

## Publisher’s note

All claims expressed in this article are solely those of the authors and do not necessarily represent those of their affiliated organizations, or those of the publisher, the editors and the reviewers. Any product that may be evaluated in this article, or claim that may be made by its manufacturer, is not guaranteed or endorsed by the publisher.

## Footnotes

^1^ www.credamo.com
